# Case Report: Simultaneous stereotactic re-irradiation using HyperArc for 44 brain metastases after whole-brain radiotherapy

**DOI:** 10.3389/fonc.2026.1949603

**Published:** 2026-09-24

**Authors:** Maximilian Römer, Peter Bublak, Irina Mäurer, Philipp Franke, Sonia Drozdz, Georg Wurschi, Noreen Murr, Klaus Pietschmann, Matthias Mäurer

**Affiliations:** 1 Department of Radiotherapy and Radiation Oncology, Jena University Hospital, Jena, Germany; 2 Comprehensive Cancer Center Central Germany, Jena, Germany; 3 Department of Neurology, Jena University Hospital, Jena, Germany

**Keywords:** brain metastases, case report, HyperArc, neurocognitive assessment, re-irradiation, small-cell lung cancer, stereotactic radiotherapy, whole-brain radiotherapy

## Abstract

**Background:**

Treatment options for disseminated intracranial recurrence after whole-brain radiotherapy (WBRT) are limited. Repeat WBRT offers modest efficacy and may worsen neurocognitive function. Modern single-isocenter techniques such as HyperArc may enable efficient stereotactic radiotherapy (SRT) of numerous brain metastases.

**Case presentation:**

A 62-year-old man with extensive-stage small-cell lung cancer and more than 40 brain metastases received systemic therapy followed by WBRT with 30 Gy in 10 fractions, resulting in near-complete intracranial regression. Approximately ten months later, magnetic resonance imaging revealed 41 new lesions and progression of 3 previously detected metastases. Because of his excellent performance status, all 44 lesions were treated with fractionated HyperArc SRT using 21.0 Gy in 3 fractions. Standardized neurocognitive testing was performed before SRT and repeated after 4 and 12 weeks. It demonstrated only selective deterioration without global or subjectively relevant impairment. Follow-up imaging after subsequent initiation of tarlatamab showed marked regression after 4 weeks and near-complete response after 3 months. Two additional lesions were subsequently treated with SRT, followed 7 months later by a second HyperArc course for 8 new metastases. At last follow-up, no treated lesion had locally recurred, and no radiographic radionecrosis was observed.

**Conclusion:**

Fractionated single-isocenter HyperArc SRT may represent a feasible salvage option for carefully selected patients with extensive intracranial recurrence after WBRT. The observed local control and limited neurocognitive deterioration support further prospective evaluation of this approach.

## 1 Introduction

Brain metastases (BMs) are common and affect 10%-40% of all patients with solid tumors (1). For patients with more than 10 BMs, whole-brain radiotherapy (WBRT) is still a commonly used treatment option and remains recommended or considered in international guidelines, particularly when focal treatment is not feasible (2). However, WBRT is associated with long-term neurocognitive toxicity (3). A possible alternative is the use of stereotactic radiotherapy (SRT), as it offers high local control and may better preserve neurocognition (4, 5). These advantages have led to SRT being the treatment of choice for patients with limited BMs (2). Evidence for the safety and efficacy of SRT for extensive BMs remains sparse and is being evaluated prospectively, including NCT04891471. Thus, SRT is often withheld in favor of WBRT because of concerns regarding out-of-field recurrence and radionecrosis.

The situation is even more complex for patients receiving re-irradiation, especially after previous WBRT, as there are currently no guidelines explicitly focusing on the re-irradiation of BMs. Furthermore, the current evidence is limited, especially for patients with disseminated cerebral recurrence. While studies evaluating repeat WBRT have reported relatively short median survival times of 2–7 months and limited treatment efficacy (6–9), studies investigating SRT have shown median overall survival (OS) ranging from 6 to 18 months (10, 11). Moreover, SRT typically causes less neurocognitive decline (12). However, treating numerous lesions can be limited by complex planning and prolonged delivery. These problems may be mitigated by modern single-isocenter linear accelerator-based techniques, such as HyperArc (Varian, Palo Alto, CA, USA). The technology allows for efficient treatment planning and rapid treatment delivery (13). We report a case of a patient with disseminated cerebral recurrence with >10 BMs after WBRT treated with fractionated SRT using HyperArc technology.

## 2 Case presentation

A 62-year-old man with newly diagnosed extensive-stage SCLC was referred to our center for management of multiple brain metastases after completing three cycles of first-line carboplatin, etoposide, and atezolizumab. Initial staging with fluorodeoxyglucose positron emission tomography/computed tomography (FDG-PET/CT) had shown a large hypermetabolic pulmonary mass and widespread extracranial metastatic disease, while brain magnetic resonance imaging (MRI) demonstrated more than 40 supra- and infratentorial metastases. Histopathological examination of a bronchoscopic biopsy had confirmed SCLC, classified as cT4 cN3 cM1c2, stage IVB, according to the ninth edition of the International Association for the Study of Lung Cancer Tumor-Node-Metastasis classification. The overall clinical course, including systemic treatment and radiotherapy courses, is summarized in **Figure 1**.

**Figure 1:**
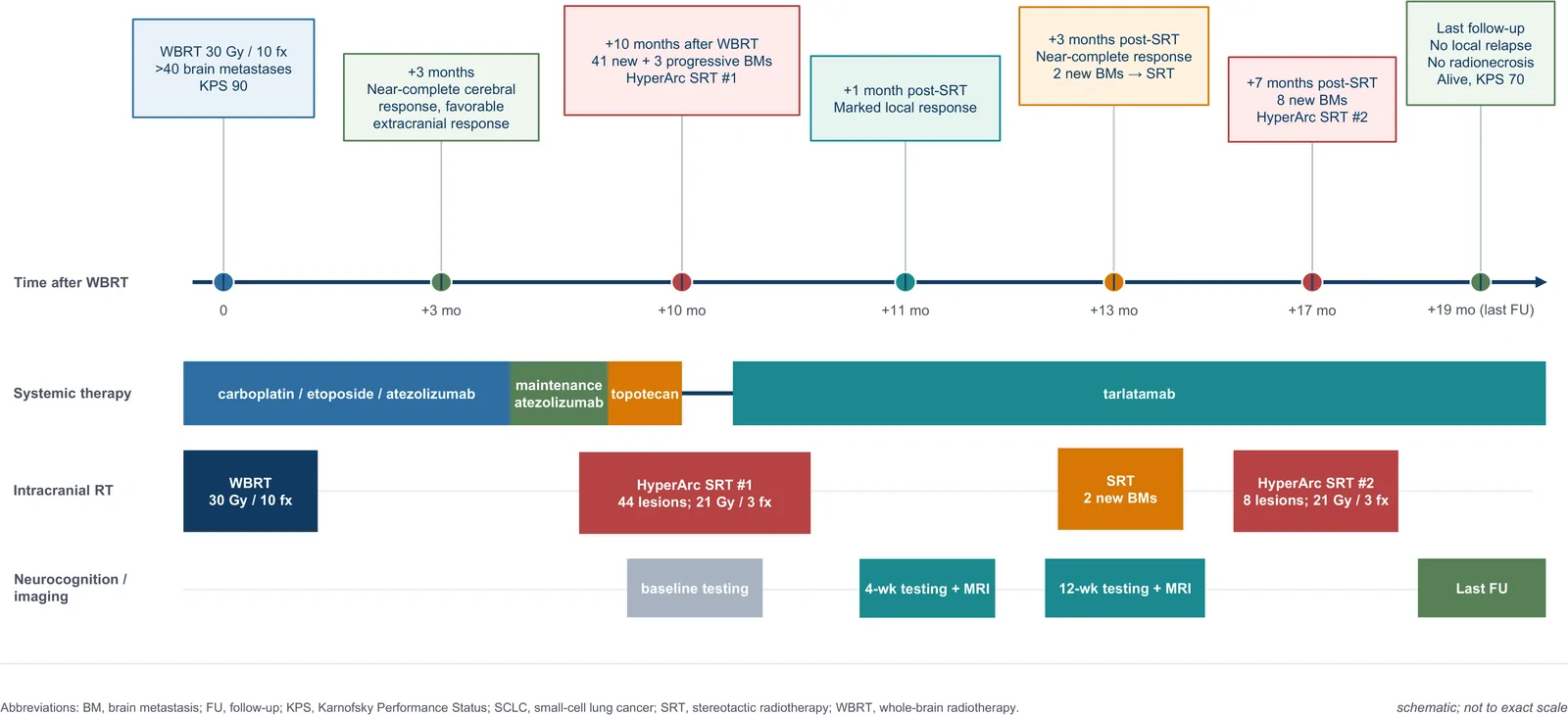
Timeline of the patient’s clinical course and treatment. The timeline summarizes the sequence of systemic therapies, radiotherapy treatment courses, intracranial response and progression, neurocognitive assessments, and radiological follow-up. Time intervals are not drawn to scale. BM, brain metastasis; FU, follow-up; fx, fractions; KPS, Karnofsky Performance Status; mo, months; MRI, magnetic resonance imaging; RT, radiotherapy; SRT, stereotactic radiotherapy; WBRT, whole-brain radiotherapy; wk, weeks.

At the time of the initial SCLC diagnosis, he had also been diagnosed with bilateral pulmonary embolism involving nearly all segmental branches of the right pulmonary artery and the left S6 branch, for which therapeutic anticoagulation was initiated. Apart from ongoing tobacco use with a cumulative exposure of more than 30 pack-years, he had no other relevant comorbidities or family history of cancer. His general condition was excellent, with a Karnofsky Performance Status (KPS) of 90. The patient had neither focal neurological deficits nor subjective cognitive symptoms. Given the multiple brain metastases and the small-cell histology, we performed WBRT with a total dose of 30 Gray (Gy) in 10 fractions. Due to the proximity of the metastases to both hippocampi, we performed WBRT without hippocampal avoidance. Treatment was delivered as planned without treatment interruptions and was well tolerated with no acute toxicity. Afterwards, systemic therapy was continued. After a total of six cycles, restaging demonstrated a favorable extracranial response. Follow-up MRI 3 months after WBRT additionally showed near-complete regression of the cerebral metastases. Maintenance therapy with atezolizumab was subsequently initiated, but had to be terminated within less than two months due to progressive mediastinal and pulmonary disease necessitating a treatment switch to topotecan.

Nine months after WBRT, brain MRI revealed three new intracranial lesions, each measuring around 4 mm in diameter, alongside extracranial progression. Topotecan was subsequently terminated. At re-evaluation, the patient again had an excellent general condition, with a KPS of 90. Neuronavigation MRI performed 5 weeks later using a protocol comparable to the previous MRI showed 41 new metastases and progression of the 3 known lesions, distributed supra- and infratentorially with a maximum diameter of 8 mm and without leptomeningeal disease, consistent with rapid intracranial progression. Owing to the patient’s excellent performance status and following multidisciplinary review, stereotactic radiotherapy to all detectable intracranial lesions was favored over repeat WBRT or systemic therapy alone. Due to rapid intracranial progression, staged SRT was considered unsuitable. Simulation for brain SRT was performed in the supine position with a rigid thermoplastic mask (Qfix, Avondale, PA, USA). Radiotherapy was delivered using HyperArc technology on a Varian TrueBeam linear accelerator (Varian, Palo Alto, CA, USA) with 21.0 Gy in 3 fractions. We used a robotic couch with six degrees of freedom, and daily image guidance with the ExacTrac (Brainlab, Munich, Germany) system, which uses stereoscopic X-ray imaging. Because four metastases were close to the optic pathway, including two directly adjacent lesions, organ-at-risk constraints adjusted for prior WBRT were prioritized over full planning target volume (PTV) coverage for these lesions. Key dosimetric parameters are summarized in **Table 1** with additional details in **Supplementary Material 1**. A three-dimensional visualization of the spatial distribution of all 44 PTVs is provided in **Supplementary Material 2**. Two weeks after radiotherapy, tarlatamab was initiated.

**Table 1:** Treatment and dosimetric characteristics of the initial HyperArc SRT.

Treatment and dosimetric parameters	Value
Number of lesions	44
Total dose	21.0 Gy
Dose per fraction	7.0 Gy
Prescription	80% isodose
Total GTV	4.15 cm³
Total PTV	34.91 cm³
PTV margin	3 mm
PTV coverage	93.3%
GTV coverage	97.6%
PTV coverage of the 42 metastases not directly adjacent to the optic pathway	95.9%
GTV coverage of the 42 metastases not directly adjacent to the optic pathway	99.7%
Brainstem D1%	9.20 Gy
Optic chiasm D1%	6.07 Gy
Left optic nerve D1%	5.42 Gy
Right optic nerve D1%	6.01 Gy
Brain mean dose	12.1 Gy
Brain V20Gy	140 cm³
Brain V21Gy	91 cm³
Treatment time	13.5 min/fraction
Beam-on time	3.5 min/fraction

Before treatment, the patient underwent extensive neurocognitive testing. These tests were repeated 4 weeks and 12 weeks after treatment. To avoid practice effects, parallel test versions were used for the assessment of memory functions and verbal fluency. The results are presented in **Figure 2**. The neurocognitive profile indicates selective post-therapy deterioration rather than global cognitive decline. The most relevant changes were observed in visuospatial memory, particularly visuospatial learning, and in executive functioning (i.e., inhibition as assessed by color-word-interference). Visual processing speed, as measured by the Symbol Digit Modalities Test, also showed a moderate decline. In contrast, verbal delayed recall, semantic fluency, and visuomotor speed measured with Trail Making A, as well as Stroop color naming, remained largely stable. Verbal learning and lexical fluency were already impaired at baseline, making therapy-associated change more difficult to interpret, but they appeared generally unchanged. Thus, selective neurocognitive deterioration was detectable 4 weeks after SRT and remained largely stable at 12 weeks. Decline was mostly confined to attentional and executive aspects of cognition, while more rapid forgetting, a typical symptom of hippocampal damage, was not observed. Further details on the neurocognitive testing procedures and results are presented in **Supplementary Material 3**. Also, the patient did not report any subjective neurocognitive changes or difficulties in everyday functioning.

**Figure 2:**
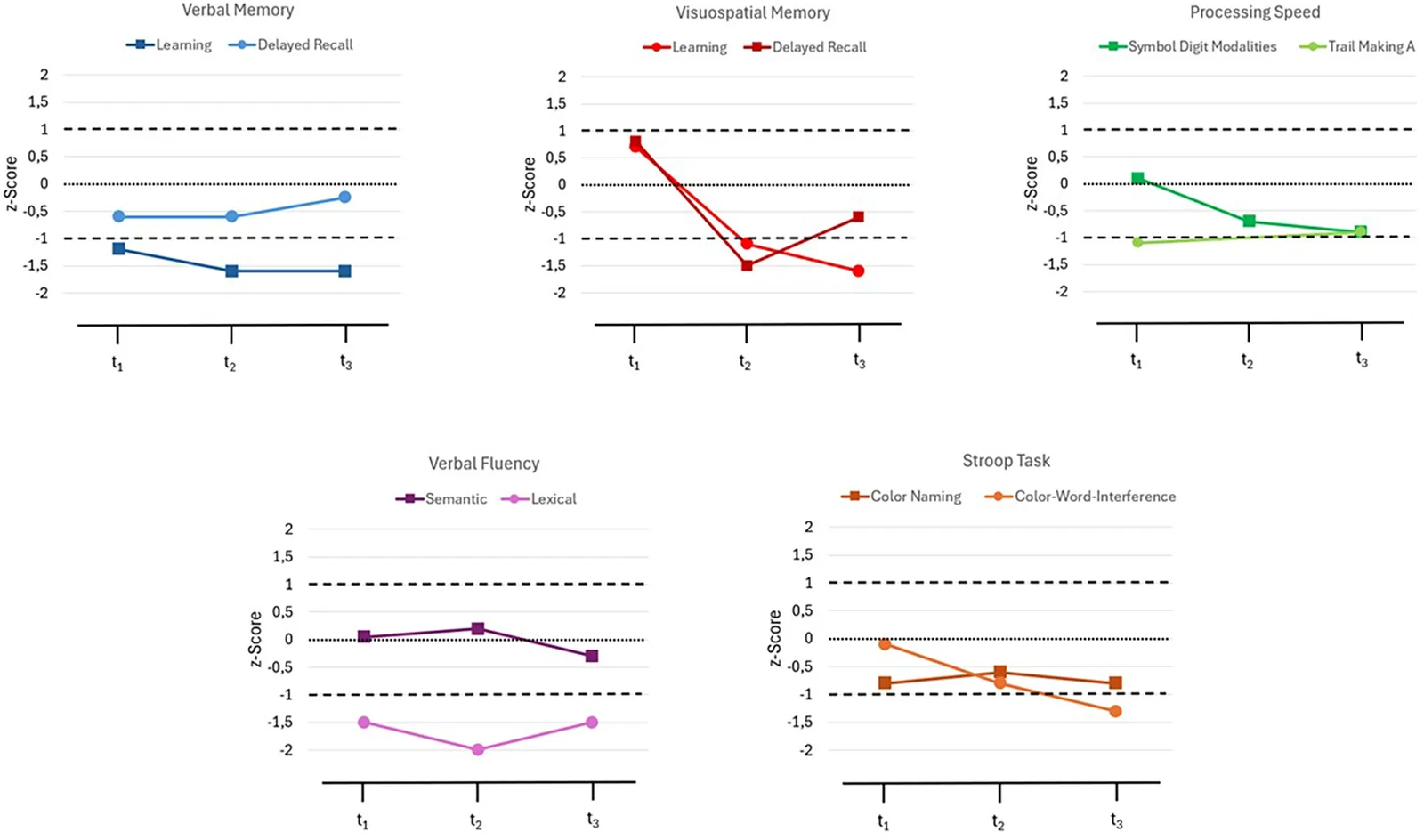
Results of the neurocognitive assessment. Neurocognitive performance is displayed as z-scores at baseline before stereotactic radiotherapy (t_1_), 4 weeks after treatment (t_2_), and 12 weeks after initial HyperArc SRT (t_3_). The assessment included the verbal learning and memory test, brief visuospatial memory test – revised, symbol digit modalities test, trail making test, stroop color–word test, semantic verbal fluency test, and phonemic verbal fluency test. Trail making a was not available at t_2_. The dotted horizontal line indicates the normative mean (z = 0), and dashed horizontal lines indicate ±1 standard deviation. Negative z-scores represent performance below the normative mean.

Follow-up MRI approximately 4 weeks after SRT demonstrated an excellent local response, with a marked reduction in lesion size. Repeat imaging 3 months after SRT showed a near-complete response of all irradiated lesions. Serial MRI examinations were reviewed by experienced neuroradiologists with individual assessment of all treated lesions, but no predefined response criteria were applied. Representative MRI slices are shown in **Figure 3**. However, two new intracranial lesions measuring 8 and 4 mm developed and were treated with SRT. During follow-up, tarlatamab was continued without evidence of neurological or other toxicity, including cytokine release syndrome, and without the need for treatment interruptions or dose reduction. Approximately 7 months after the initial SRT, 8 additional intracranial lesions developed, requiring treatment with another HyperArc SRT course of 21.0 Gy in 3 fractions, which was again well tolerated. SRT courses following the initial HyperArc SRT involved metastases sufficiently separated from previously irradiated targets and critical organs, allowing full target coverage without relevant dosimetric compromise. No local recurrence was observed among the initial 44 lesions during follow-up. The subsequently treated lesions also remained locally controlled. At the last follow-up, approximately two months after the final SRT course, there was no radiographic evidence of radionecrosis; the patient was alive, with a KPS of 70, and reported no neurocognitive impairment in everyday functioning. Toxicity was regularly assessed according to the Common Terminology Criteria for Adverse Events (CTCAE) v5.0, with no radiotherapy-related toxicity observed.

**Figure 3:**
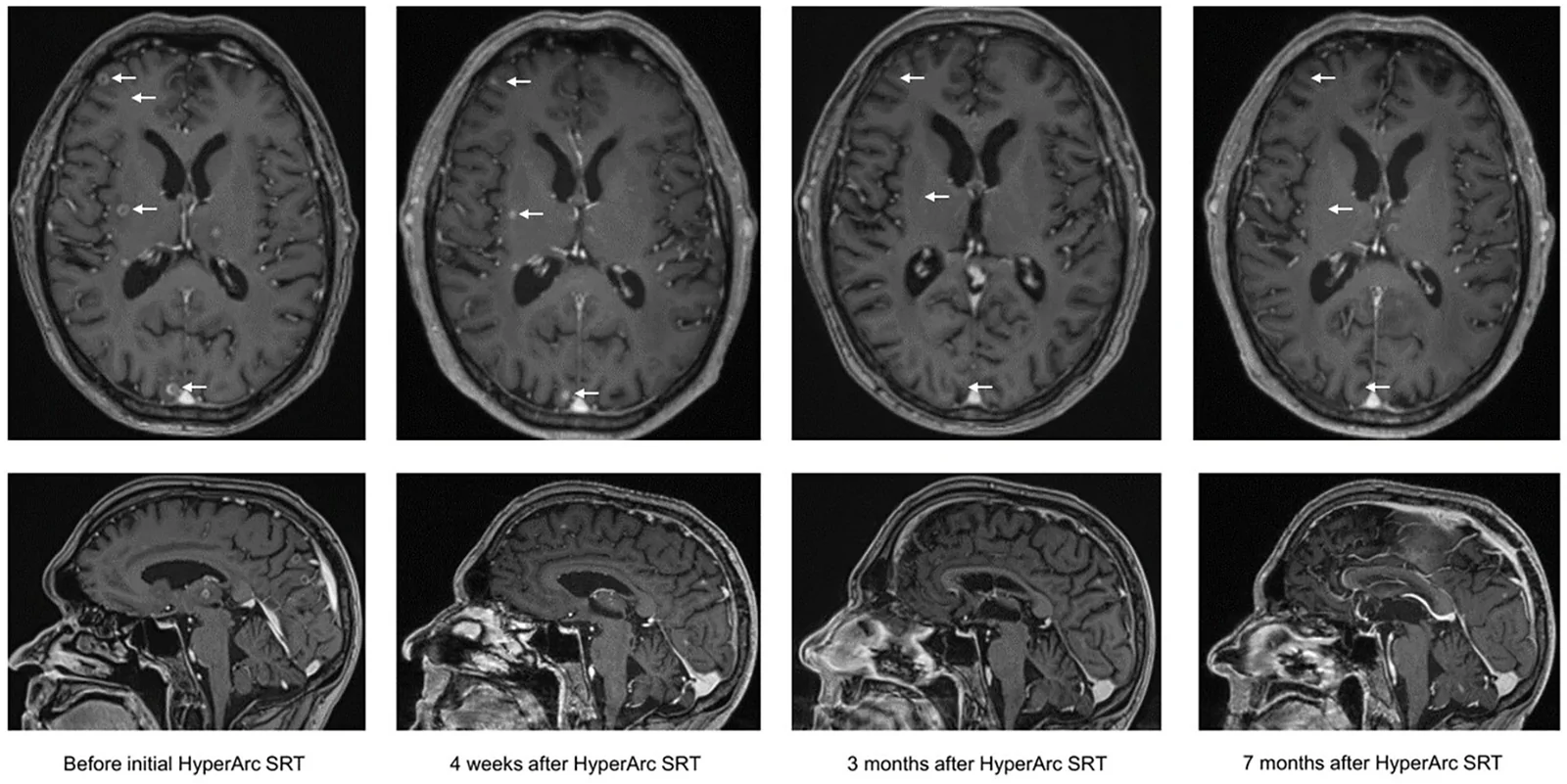
Radiographic response of the cerebral metastases. Representative contrast-enhanced T1-weighted MRI images before initial HyperArc SRT and at 4 weeks, 3 months, and 7 months after treatment. Axial images are shown in the upper row and sagittal images in the lower row. White arrows indicate representative treated metastases on the axial images. Serial imaging demonstrated marked regression and sustained local control of the irradiated lesions despite subsequent distant intracranial progression.

## 3 Discussion

This case illustrates that fractionated HyperArc SRT may be feasible as salvage treatment in carefully selected patients with disseminated intracranial recurrence after WBRT. Despite 44 lesions, prior WBRT, and rapid intracranial progression, treatment was technically feasible, rapidly delivered without treatment interruptions, and well tolerated. Historically, patients with disseminated intracranial recurrence after WBRT have often been treated with repeat WBRT or best supportive care (2, 14). However, repeat WBRT is generally associated with limited intracranial efficacy and poor survival, while also carrying a relevant risk of further neurocognitive impairment. In selected patients with preserved performance status, limited total tumor volume, and small lesions amenable to safe stereotactic treatment, focal salvage treatment may therefore represent an attractive alternative. Conversely, this approach is unlikely to be appropriate for patients with poor performance status, high intracranial tumor volume, or leptomeningeal disease. Its broader implementation also requires appropriate stereotactic planning, image guidance, and close MRI surveillance. Trials investigating SRT for recurrent brain metastases tend to report superior median OS compared with repeat WBRT, although cross-study comparisons are strongly confounded by patient selection (10). Furthermore, SRT typically causes less neurocognitive decline (12). Additionally, recent studies support SRT for selected patients with numerous BMs, including prospective data in SCLC (15, 16). However, these address upfront treatment, whereas evidence for salvage SRT for numerous BMs after WBRT remains limited.

Despite the relatively large volume of brain receiving high-dose re-irradiation, no radiographic radionecrosis was observed during the limited follow-up. Moreover, no local failures were observed in the irradiated lesions, although subsequent tarlatamab may have contributed to the observed response. The second course of SRT was likewise well tolerated. Longitudinal neurocognitive testing demonstrated selective deterioration, particularly in visual processing speed, visuospatial learning, and executive functioning, without evidence of global cognitive decline. However, attribution to SRT is limited by prior WBRT, systemic disease and treatment, and potential effects of fatigue or medication. Moreover, formal testing was limited to 12 weeks. The absence of subjectively perceived impairment at later follow-up should therefore not be considered equivalent to preserved objective neurocognitive function. These findings highlight the value of objective longitudinal neurocognitive assessment in this setting. Strengths of this report include detailed dosimetric characterization, serial high-resolution MRI follow-up, and standardized longitudinal neurocognitive testing using parallel test versions to reduce practice effects. The main limitations are the single-patient design, the inability to separate the contribution of SRT from that of tarlatamab to the observed radiological response, absence of a comparator, and limited follow-up for delayed radionecrosis and long-term neurocognitive outcomes.

## 4 Conclusions

Although no general conclusions can be drawn from a single case, our observation supports the prospective evaluation of fractionated single-isocenter SRT as a salvage approach in carefully selected patients with extensive intracranial recurrence after WBRT.

## 5 Patient perspective

Throughout the treatment course, the patient remained optimistic and expressed a high level of satisfaction with SRT. His primary motivation for choosing SRT was to pursue the treatment option offering the greatest possible chance of longer intracranial disease control. Despite detailed counselling about the substantial risk of radionecrosis associated with re-irradiation, he reported no particular fears or concerns regarding the treatment. He was grateful for the subjectively very good tolerability and did not perceive any relevant deterioration in cognitive function or everyday functioning following SRT. He stated that he would choose the same treatment again.

## Data Availability

The original contributions presented in the study are included in the article/**Supplementary Material**. Further inquiries can be directed to the corresponding author.
