# Supplementary material for: Case Report: Simultaneous stereotactic re-irradiation using HyperArc for 44 brain metastases after whole-brain radiotherapy
Source: Front Oncol. 2026 Sep 24;16:1949603. doi: 10.3389/fonc.2026.1949603 (PMC13645520; doi:10.3389/fonc.2026.1949603)
Supplement: Supplementary file 1 [file DataSheet1.pdf]

CARE Checklist of information to include when writing a case report

CARE case report guidelines

(cc) BY-NC-ND

Topic	Item	Checklist item description	Reported on Line
Title	1	The diagnosis or intervention of primary focus followed by the words "case report"	Lines 1–2
Key Words	2	2 to 5 key words that identify diagnoses or interventions in this case report, including "case report"	Lines 16–17
Abstract (no references)	3a	Introduction: What is unique about this case and what does it add to the scientific literature?	Lines 22–25, 37–40
	3b	Main symptoms and/or important clinical findings	Lines 26–36
	3c	The main diagnoses, therapeutic interventions, and outcomes	Lines 26–36
	3d	Conclusion—What is the main "take-away" lesson(s) from this case?	Lines 37–40
Introduction	4	One or two paragraphs summarizing why this case is unique (**may include references**)	Lines 42–62
Patient Information	5a	De-identified patient specific information.	Lines 64–79
	5b	Primary concerns and symptoms of the patient.	Lines 74–79
	5c	Medical, family, and psycho-social history including relevant genetic information	Lines 74–78
	5d	Relevant past interventions with outcomes	Lines 64–66, 80–87
Clinical Findings	6	Describe significant physical examination (PE) and important clinical findings.	Lines 78–79, 88–93, 137–140
Timeline	7	Historical and current information from this episode of care organized as a timeline	Lines 72–73; Figure 1
Diagnostic Assessment	8a	Diagnostic testing (such as PE, laboratory testing, imaging, surveys).	Lines 66–73, 88–93, 107–126, 135–140; Figures 2–3
	8b	Diagnostic challenges (such as access to testing, financial, or cultural)	NA – no relevant diagnostic challenges
	8c	Diagnosis (including other diagnoses considered)	Lines 66–71, 88–93
	8d	Prognosis (such as staging in oncology) where applicable	Lines 69–72, 84–93, 123–140
Therapeutic Intervention	9a	Types of therapeutic intervention (such as pharmacologic, surgical, preventive, self-care)	Lines 64–66, 80–87, 94–106, 127–140
	9b	Administration of therapeutic intervention (such as dosage, strength, duration)	Lines 64–66, 80–83, 97–106, 127–140; Table 1
	9c	Changes in therapeutic intervention (with rationale)	Lines 85–96, 106, 127–132
Follow-up and Outcomes	10a	Clinician and patient-assessed outcomes (if available)	Lines 84–85, 107–140, 178–185; Figures 2–3
	10b	Important follow-up diagnostic and other test results	Lines 84–85, 107–140; Figures 2–3
	10c	Intervention adherence and tolerability (How was this assessed?)	Lines 82–83, 128–140, 179–185
	10d	Adverse and unanticipated events	Lines 82–83, 128–140, 159–160
Discussion	11a	A scientific discussion of the strengths AND limitations associated with this case report	Lines 169–173
	11b	Discussion of the relevant medical literature **with references**.	Lines 142–173
	11c	The scientific rationale for any conclusions (including assessment of possible causes)	Lines 142–173
	11d	The primary "take-away" lessons of this case report (without references) in a one paragraph conclusion	Lines 174–177
Patient Perspective	12	The patient should share their perspective in one to two paragraphs on the treatment(s) they received	Lines 178–185
Informed Consent	13	Did the patient give informed consent? Please provide if requested	Yes ☒ No ☐
