# Supplementary material for: Case Report: Simultaneous stereotactic re-irradiation using HyperArc for 44 brain metastases after whole-brain radiotherapy
Source: Front Oncol. 2026 Sep 24;16:1949603. doi: 10.3389/fonc.2026.1949603 (PMC13645520; doi:10.3389/fonc.2026.1949603)
Supplement: Supplementary file 2 [file Table1.docx]

**Supplement 1.** Supplementary dosimetric information for the index HyperArc SRT course

**Treatment context.** The index HyperArc course treated 44 brain metastases with 21 Gy in 3 fractions after prior whole-brain radiotherapy (WBRT) with 30 Gy in 10 fractions. The normal brain structure used for dose-volume evaluation was defined as whole brain minus GTV. Four metastases were close to the optic pathway. For two lesions, the PTV approached within 1 mm of the optic pathway; OAR constraints accounting for prior WBRT were prioritized over complete target coverage. The lower aggregate PTV/GTV coverage when all 44 lesions are included reflects this deliberate undercoverage.

**Table A:** Target and normal-brain dosimetry

| **Parameter** | **Value** |
| --- | --- |
| Number of treated lesions | 44 |
| Largest metastasis | 0.25 cm³; maximum diameter 0.8 cm |
| Total GTV | 4.15 cm³ |
| Total PTV | 34.91 cm³ |
| PTV margin | 3 mm |
| Prescription | 21.0 Gy in 3 fractions; 80% isodose |
| PTV coverage, all 44 lesions | 93.3% |
| GTV coverage, all 44 lesions | 97.6% |
| PTV coverage excluding the 2 lesions closest to the optic pathway | 95.9% |
| GTV coverage excluding the 2 lesions closest to the optic pathway | 99.7% |
| Normal-brain volume (brain − GTV) | 1568.04 cm³ |
| Normal-brain mean dose | 12.1 Gy |
| Normal-brain V20Gy | 140 cm³ |
| Normal-brain V21Gy | 91 cm³ |

**Table B:** Organ-at-risk dosimetry for the index HyperArc course

| **Structure** | **Volume (cm³)** | **Mean dose (Gy)** | **D2% (Gy)** | **D1% (Gy)** |
| --- | --- | --- | --- | --- |
| Brainstem | 25.39 | 7.651 | 9.005 | 9.199 |
| Optic chiasm | 0.65 | 4.897 | 5.910 | 6.069 |
| Left optic nerve | 0.88 | 3.603 | 5.282 | 5.422 |
| Right optic nerve | 1.02 | 3.465 | 5.081 | 6.008 |
| Left cochlea | 1.52 | 14.037 | 18.120 | 18.410 |
| Right cochlea | 1.44 | 7.717 | 8.981 | 9.132 |
| Left hippocampus | 2.93 | 8.820 | 14.285 | 14.543 |
| Right hippocampus | 2.78 | 10.684 | 15.508 | 15.985 |
| Left lens | 0.10 | 2.682 | 3.522 | 3.653 |
| Right lens | 0.14 | 5.297 | 7.639 | 7.684 |

**Table C:** Approximate cumulative OAR dose context after prior WBRT

| **Structure** | **Index SRT D1% (Gy)** | **Approx. cumulative physical dose* (Gy)** | **Approx. cumulative BED₂* (Gy₂)** | **Approx. cumulative EQD2₂* (Gy)** |
| --- | --- | --- | --- | --- |
| Brainstem | 9.199 | 39.2 | 98.3 | 49.2 |
| Optic chiasm | 6.069 | 36.1 | 87.2 | 43.6 |
| Left optic nerve | 5.422 | 35.4 | 85.3 | 42.7 |
| Right optic nerve | 6.008 | 36.0 | 87.0 | 43.5 |

For contextual estimation, each listed OAR was assumed to have received the full prescribed WBRT dose of 30 Gy in 10 fractions. BED and EQD2 were calculated using the linear-quadratic model with α/β = 2 Gy and the D1% of the index SRT plan as the near-maximum re-irradiation dose. These values do not represent voxel-wise accumulated dose and do not account for spatial dose heterogeneity or tissue recovery between courses.

**Table D:** Target coverage in the vicinity of the optic pathway

| **Region** | **Target** | **Volume (cm³)** | **D99 (Gy)** | **D98 (Gy)** | **D95 (Gy)** | **Mean dose (Gy)** | **D50 (Gy)** |
| --- | --- | --- | --- | --- | --- | --- | --- |
| Left optic pathway | PTV28 | 2.44 | 10.20 | 11.63 | 13.64 | 19.68 | 20.18 |
|  | GTV27 | 0.12 | 20.02 | 20.09 | 20.57 | 22.68 | 22.81 |
|  | GTV37 | 0.03 | 16.69 | 16.69 | 17.66 | 19.25 | 19.26 |
| Right optic pathway | PTV26 | 3.74 | 4.86 | 5.20 | 7.11 | 19.57 | 21.85 |
|  | GTV24 | 0.12 | 21.02 | 21.69 | 21.93 | 22.69 | 22.74 |
|  | GTV25 | 0.05 | 10.76 | 10.81 | 10.85 | 15.35 | 14.92 |
|  | GTV36 | 0.09 | 22.69 | 22.71 | 22.74 | 23.52 | 23.67 |

Several metastases in close proximity to the optic pathway were incorporated into composite PTVs. PTV28 encompassed GTV27 and GTV37 in the region of the left optic nerve, whereas PTV26 encompassed GTV24, GTV25, and GTV36 in the region of the right optic nerve. Consequently, PTV-level dose-volume parameters do not directly reflect the coverage of each individual metastasis. To comply with the predefined clinical goals accounting for the previous WBRT (Dmax 7 Gy for both optic nerves), reduced target coverage was deliberately accepted in these regions. PTV28 had a D95 of 13.64 Gy and PTV26 a D95 of 7.11 Gy. Analysis of the individual GTVs showed that undercoverage was largely confined to GTV37 and GTV25, whose corresponding PTVs approached within 1 mm of the optic pathway. Their D95 values were 17.66 Gy and 10.85 Gy, respectively. All predefined organs-at-risk clinical goals were met.
