# Supplementary material for: Case Report: Simultaneous stereotactic re-irradiation using HyperArc for 44 brain metastases after whole-brain radiotherapy
Source: Front Oncol. 2026 Sep 24;16:1949603. doi: 10.3389/fonc.2026.1949603 (PMC13645520; doi:10.3389/fonc.2026.1949603)
Supplement: Supplementary file 3 [file Table2.docx]

**Supplement 3**. Supplementary neurocognitive testing results

Verbal memory was assessed with the „Verbaler Lern- und Merkfähigkeitstest“ (VLMT; Helmstaedter et al., 2001 [1]), a German equivalent of the Rey Auditory Verbal Learning test (RAVLT). The VLMT provides 3 parallel versions of word lists (lists A, C, and D), each comprising a different set of 15 words. The variable “learning” was operationalised by the sum of correctly reported words across the five learning trials (max. 75 words). The variable “delayed recall” was operationalised by the number of words reported in the free recall trial after 30 minutes (max. 15 words). Age-adjusted normative data were taken from the paper published by Volz-Sidiropoulou et al. (2010) [2].

Visuospatial memory was assessed with the “Brief Visuospatial Memory Test-Revised” (BVMT-R; Benedict, 1997 [3]) which provides 6 parallel versions of visuospatial stimulus arrays. Forms 1, 2 and 3 were used. The variable “learning” was operationalised by the sum of the points achieved in the learning trials 1-3 (max. 36 points). The variable “delayed recall” was operationalised by the points achieved in the free recall trial after a delay of 30 minutes (max. 12 points). Age-adjusted normative data were taken from the test manual.

Processing speed was assessed by two procedures: The Symbol Digit Modalities Test (SDMT; Smith, 2013 [4]) which assesses the number of correctly reported digits in 90 seconds. Age-adjusted norms were taken from the manual. The Trail Making Test, Version A, (TMT-A) is part of the CERAD test battery (Schmid et al., 2014 [5]) and assesses the time (in seconds) required to connect irregularly arranged circles, numbered from 1 to 25, on a sheet of paper, with a pencil in ascending order. The test provides normative data adjusted for age, sex, and education. [6]

Verbal fluency was assessed with the “Regensburger Wortflüssigkeitstest” (RWT; Aschenbrenner, Tucha and Lange, 2001 [7]). The semantic condition demands naming as many members of a given category as possible, within 1 minute. Categories used in the 3 different assessments were “animals”, “groceries”, and “forenames”. The lexical condition requires generating as many words as possible with a given initial letter within 1 minute, avoiding proper names, repetitions, and using the same root word with different endings. Letters “M”, “K”, and “P” were used in the 3 different assessments. The test provides age-adjusted norms, taken from the manual.

Finally, the Stroop colour word interference test was used to assess inhibitory control. The specific procedure applied was a subtest of the “Nürnberger-Alters-Inventar” (NAI; Oswald and Fleischmann, 1999 [8]). The colour condition assesses the time required to correctly name the colour of 36 colour bars arranged in a 6x6 array. The interference condition assesses the time required to correctly name the font colour of colour words with a divergent meaning, also arranged in a 6x6 array. The difference between the interference and the colour condition defines the interference effect. The test manual provides age-adjusted norms.

Clinically meaningful deterioration was defined as an RCI ≤ −1.96 for measures in which higher scores indicate better performance and as an RCI ≥ +1.96 for measures in which higher values indicate poorer performance. The cut-off was calculated as:

Cut-off = 1,96 x SD √(2(1-r)),

where SD is the standard deviation in the normative sample, and r is the reliability coefficient of the respective test. Testing was performed in German. Education-adjusted norms were used where provided by the respective normative system.

For each test instrument, Table A provides the alternative test versions used at the 3 different assessment times, the raw scores, reliable change indices, and cut-off scores representing the clinically meaningful deterioration.

| Table A: Raw scores, reliable change indices (RCI), and clinically meaningful difference (CMD) scores for each assessment instrument. | | | | | | |
| --- | --- | --- | --- | --- | --- | --- |
| **Domain** | **Test** | **Measure** | **Time** | **Raw score** | **RCI** | **CMD** |
| Verbal Memory | VLMT | Learning trials 1-5,  sum of words | t_1_ | 40 | - | *−12* |
|  |  |  | t_2_ | 38 | *−0.34* |  |
|  |  |  | t_3_ | 38 | *−0.33* |  |
|  |  | Delayed recall,  number of words | t_1_ | 9 | - | *−3* |
|  |  |  | t_2_ | 9 | *0* |  |
|  |  |  | t_3_ | 10 | *0.63* |  |
| Visuospatial Memory | BVMT-R | Learning trials 1-3,  points | t_1_ | 26 | - | *−7* |
|  |  |  | t_2_ | 16 | *−3.10*** |  |
|  |  |  | t_3_ | 13 | *−4.03**** |  |
|  |  | Delayed recall,  points | t_1_ | 10 | - | *−2* |
|  |  |  | t_2_ | 5 | *−5.51**** |  |
|  |  |  | t_3_ | 7 | *−3.31**** |  |
| Processing speed | SDMT | Correctly reported digits, number | t_1_ | 46 | - | *−13* |
|  |  |  | t_2_ | 39 | *−1.05* |  |
|  |  |  | t_3_ | 37 | *−1.35* |  |
|  | TMT-A | Time, seconds | t_1_ | 57 | - | *20^#^* |
|  |  |  | t_2_ | n.a. | - |  |
|  |  |  | t_3_ | 53 | *−0.32* |  |
| Verbal Fluency | RWT | Semantic, number of words | t_1_ | 20 | - | - |
|  |  |  | t_2_ | 20 | *n.c.* |  |
|  |  |  | t_3_ | 19 | *n.c.* |  |
|  |  | Lexical, number of words | t_1_ | 7 | - | - |
|  |  |  | t_2_ | 6 | *n.c.* |  |
|  |  |  | t_3_ | 4 | *n.c.* |  |
| Inhibition | Stroop Task | Color naming  time, seconds | t_1_ | 29 | - | *9^#^* |
|  |  |  | t_2_ | 27 | *−0.47* |  |
|  |  |  | t_3_ | 29 | 0 |  |
|  |  | Interference  time difference, seconds | t_1_ | 19 | - | *14^#^* |
|  |  |  | t_2_ | 28 | *1.27* |  |
|  |  |  | t_3_ | 35 | *2.26** |  |
| VLMT: Verbaler Lern- und Merkfähigkeitstest (t_1_: Word List A; t_2_: List C; t_3_: List D);  BVMT-R: Brief Visuospatial Memory Test-Revised (t_1_: Stimulus Set 1; t_2_: Set 2; t_3_: Set 3);  SDMT: Symbol Digit Modalities Test; TMT-A: Trail Making Test, Version A;  RWT: Regensburger Wortflüssigkeitstest (Semantic Condition: t_1_: Animals; t_2_: Groceries; t_3_: Fore­names; Lexical Condition: t_1_: Letter M; t_2_: Letter K; t_3_: Letter P);  t_1_: Pre-test; t_2_: first follow-up; t_3_: second follow-up; n.a.: not assessed; n.c.: not computable.  **p < .05; **p < .01; ***p < .001*.  ^#^In these tests, a deterioration is reflected by positive values. | | | | | | |

**References**

1.	Helmstaedter, C., M. Lendt, and S. Lux, *Verbaler Lern- und Merkfähigkeitstest: VLMT; Manual*. 2001, Göttingen: Beltz Test. 81.

2.	Volz-Sidiropoulou, E., et al., *Erweiterte Altersnormen zum Verbalen Lern- und Merkfähigkeitstest (VLMT).* Klinische Diagnostik und Evaluation, 2010. **3**(3): p. 226-243.

3.	Benedict, R.H.B., *Brief Visuospatial Memory Test-Revised: Professional Manual*. 1997, Lutz, FL: Psychological Assessment Resources, Inc.

4.	Smith, A., *Symbol Digit Modalities Test*. 13th edition ed. 2013, Los Angeles, CA: Western Psychological Services.

5.	Schmid, N.S., et al., *The Extension of the German CERAD Neuropsychological Assessment Battery with Tests Assessing Subcortical, Executive and Frontal Functions Improves Accuracy in Dementia Diagnosis.* Dementia and Geriatric Cognitive Disorders Extra, 2014. **4**(2): p. 322-334. doi: 10.1159/000357774

6.	Memory, C. *CERAD-Plus Online*. Memory Clinic 2026/09/05; Available from: https://www.memoryclinic.ch/de/main-navigation/neuropsychologen/cerad-plus/auswertungprogramm/cerad-plus-online.

7.	Aschenbrenner, S., O. Tucha, and K.W. Lange, *Regensburger Wortflüssigkeits-Test: RWT; Handanweisung*. 2001, Göttingen: Hogrefe, Verlag für Psychologie. 121.

8.	Oswald, W.D. and U.M. Fleischmann, *Nürnberger-Alters-Inventar (NAI): NAI-Testmanual und -Textband*. 4., unveränderte Auflage ed. 1999, Göttingen: Hogrefe, Verlag für Psychologie.
